# Efficient real time small object detection framework in aerial images using edge awareness and dynamic convolution

**DOI:** 10.1038/s41598-026-53433-3

**Published:** 2026-05-22

**Authors:** Tieshan Zhang, Shaoyuan Xi, Dongyue Chen, Zhong Ren

**Affiliations:** 1 College of Information Science and Engineering, Northeastern University ShenYang, LiaoNing, 110819 China; 2 Department of Electrical Engineering, Ningxia Institute of Science and Technology ShiZuiShan, NingXia, 753000 China

**Keywords:** Small object detection, Real-time detection, Edge awareness, Dynamic convolution, Computational efficiency, Engineering, Mathematics and computing

## Abstract

Small object detection in high-resolution images remains challenging because target instances usually occupy only a limited number of pixels, making their appearance cues weak and easily overwhelmed by background noise. In addition, the repeated downsampling operations used in conventional detectors tend to suppress high-frequency edge information and fine-grained spatial details, which are crucial for accurate localization of small objects. Meanwhile, many existing methods improve detection performance by introducing heavier multi-scale feature fusion strategies or more complex prediction heads, which significantly increase computational cost, parameter redundancy, and inference latency, thereby limiting real-time deployment on resource-constrained devices. To address these issues, this paper proposes GDD-YOLO, an efficient real-time small object detection framework. Specifically, a Global Edge Information Transfer (GEIT) module is designed to extract multiscale edge cues from shallow features and propagate them across the backbone to strengthen boundary-aware localization of small objects. In addition, a Dynamic Inception Mixer (DIM) is introduced to overcome the limited scale adaptability of fixed convolution kernels by performing input-adaptive multi-branch convolutional aggregation with reduced complexity. Furthermore, a lightweight detection head (DECDH) is developed using shared convolution and detail-enhanced operators to preserve contextual and local structural information while reducing parameter overhead. Experiments on the VisDrone dataset show that GDD-YOLO achieves 26.2% AP, outperforming YOLOv11-S by 2.4%, while reducing the number of parameters by 16.8% and computational cost by 14.6%. These results demonstrate that GDD-YOLO provides an effective balance between detection accuracy and inference efficiency for real-time small object detection on edge devices.

## Introduction

Small object detection remains one of the most challenging and enduring problems in the field of computer vision. Targets in this category are typically characterized by limited pixel coverage, weak texture cues, and blurred appearances in images, making them particularly difficult to localize and classify accurately. Although deep neural networks have achieved remarkable progress in detecting large objects even under low-resolution conditions, their performance often deteriorates significantly when applied to small objects in high-resolution imagery. This challenge is further exacerbated in real-world applications that require deployment on resource-limited hardware, where achieving an optimal trade-off between efficiency and detection accuracy remains a critical and unresolved issue.

Early object detection research was dominated by two-stage frameworks, which generally decomposed the detection task into region proposal generation, region classification, and bounding-box regression. Representative methods include R-CNN^1^ and Faster R-CNN^2^, both of which achieved strong detection accuracy and established important foundations for subsequent detection studies. Nevertheless, these methods also suffered from several drawbacks, such as high computational complexity, multi-stage optimization, and relatively low inference speed. To address the efficiency issue, several improved variants were proposed, including R-FCN^3^ and Light-Head R-CNN^4^, which aimed to accelerate the detection process while maintaining competitive accuracy. On top of the Faster R-CNN architecture, Mask R-CNN^5^ introduced an additional instance segmentation branch, which alleviated the weak alignment between feature maps and the original image and further improved detection and segmentation performance. Cascade R-CNN^6^ enhanced detection quality by employing a cascade of multiple detectors with progressively stricter localization requirements. In addition, DiffusionDet^7^ extended the application of diffusion models to object detection by modeling bounding-box prediction as a progressive denoising process, where Gaussian noise perturbation and coordinate scaling were used to simulate diffusion during training, and the final boxes were obtained through iterative denoising during inference.

Overall, most two-stage detectors follow a coarse-to-fine design philosophy. The first stage primarily emphasizes candidate generation and high recall, whereas the second stage further refines object localization and classification to improve discriminative performance. Despite their high accuracy, two-stage detectors are often constrained by limited real-time capability and relatively complex architectures, which reduce their practicality in real-world engineering applications. Compared with them, one-stage object detectors provide significant advantages in terms of inference speed, computational efficiency, and ease of deployment, making them more suitable for real-time detection tasks, especially in resource-constrained scenarios.

One-stage detectors like SSD^8^ and the YOLO series^9^ streamline the detection process by performing dense predictions directly on the input image, thereby eliminating the region proposal stage. This architectural simplification substantially reduces computational overhead and enhances deployment feasibility, especially on edge devices. However, such gains in efficiency often come at the cost of reduced accuracy in detecting small objects due to insufficient feature representation and spatial detail loss.

In response, numerous studies have sought to enhance the small object detection capabilities of YOLO-family networks. For instance, Liu et al.^10^ redesigned the residual blocks in YOLOv3 and introduced hierarchical convolution enhancement units to better preserve high-frequency spatial details essential for small objects. Zhu et al.^11^ integrated a Transformer-based prediction head into TPH-YOLOv5, leveraging self-attention mechanisms to improve contextual reasoning and feature robustness. Wang et al.^12^ proposed a Cross-Layer Feature Fusion Module (FFNB) for YOLOv8 to facilitate hierarchical feature interaction and expand receptive fields, thereby improving detection in aerial imagery. Zhang et al.^13^ developed YOLO-fastest-v2 by incorporating ShuffleNet-v2 as the backbone and simplifying the Feature Pyramid Network (FPN), while Xu et al.^14^ introduced LightYOLO, which employs FasterNet blocks and a multi-scale attention mechanism to boost efficiency and discriminability.

Through continuous development and iterative refinement, the YOLO series has significantly advanced the progress of object detection technology. YOLOv7^15^, proposed by Wang et al., introduced the E-ELAN architecture, which enhanced the network’s learning capability without disrupting the original gradient propagation path. Building upon this foundation, further optimization led to the proposal of YOLOv9^16^. YOLOv9 adopted programmable gradient information and an improved efficient layer aggregation network to ensure that sufficient information was preserved in deep features. Subsequently, Wang et al. proposed YOLOv10^17^, which introduced a non-maximum-suppression-free training strategy based on consistent dual assignments, further improving the efficiency and performance of the detection framework.

In recent years, the Transformer, as a deep learning architecture built upon the attention mechanism, was initially developed for natural language processing and was later gradually introduced into computer vision tasks. ViT^18^ was the first to successfully apply the Transformer architecture to computer vision, laying the foundation for subsequent research on vision transformers. In the field of object detection, Carion et al. first proposed DETR^19^, an end-to-end detection network based on the Transformer architecture, which opened a new paradigm for object detection. Subsequently, Roh et al. proposed Sparse DETR^20^, an encoder token sparsification method designed to reduce the computational complexity of the attention mechanism in the encoder. Hou et al. argued that the slow convergence of DETR-like models mainly stemmed from the lack of structural inductive bias in the attention mechanism when processing input features, and therefore proposed Relation DETR^21^ to address this issue. However, due to their relatively high computational complexity, the DETR family has long struggled to satisfy the requirements of real-time object detection. To overcome this limitation, Zhao et al. proposed RT-DETR^22^, the first real-time object detector based on the Transformer architecture. In addition, Peng et al. introduced D-FINE^23^, which reformulated bounding-box regression as a distribution optimization problem and further improved detection performance by incorporating self-distillation techniques.

Despite these advances, the detection accuracy and robustness of existing models remain substantially challenged in high-resolution scenarios with dense small objects. Fundamental issues such as scarce spatial information, complex background interference, and high rates of false positives and false negatives continue to impede progress. These limitations underscore the need for more sophisticated architectural designs that can reconcile the competing demands of accuracy, efficiency, and generalization.

To address these challenges, this paper introduces a novel detection framework tailored for high-resolution small object detection. Our approach integrates three key components: a Global Edge Information Transfer (GEIT) module, a Dynamic Inception Mixer (DIM) module, and a lightweight detection head named DECDH (Detail-Enhanced Convolution Detection Head).

First, recognizing that precise object localization hinges on robust edge cues—which are often neglected in mainstream detectors—we design the GEIT module to explicitly extract and propagate edge information from shallow layers across all scales of the backbone network. This enables cross-scale edge enhancement and significantly improves localization precision for small objects.

Second, to overcome the limitations of conventional CNNs that employ static, fixed convolutional kernels—which struggle with the multi-scale variability and intricate appearance of small objects—we propose the DIM module. This component supports input-adaptive kernel adjustment and integrates multi-scale convolutional operations in parallel, facilitating rich cross-scale feature interaction. Built upon depthwise separable convolution, DIM substantially reduces computational cost while enhancing the network’s capacity to capture diverse and fine-grained features.

Finally, to fulfill the requirements of real-time detection on resource-constrained devices, we design a lightweight detection head (DECDH) that employs shared convolutions to minimize parameters. It further incorporates Detail-Enhanced Convolution (DEConv) to augment feature representation and generalization without compromising detection accuracy.

The main contributions of this work are summarized as follows:

### Global Edge Information Transfer (GEIT) Module:

This module systematically transfers critical edge information from shallow feature maps to deeper layers, enabling cross-scale edge-aware enhancement and significantly improving small object localization accuracy.

### Dynamic Inception Mixer (DIM) Module:

By replacing traditional static convolution with an input-adaptive, multi-scale mixing mechanism, DIM enhances the model’s ability to capture complex small object features while maintaining low computational complexity through the use of depthwise separable convolution.

### Lightweight Detection Head (DECDH):

Designed for efficient inference, this head utilizes shared convolutions and Detail-Enhanced Convolution (DEConv) to drastically reduce parameter counts and computational overhead while preserving high detection performance.

Collectively, these innovations provide a balanced and effective solution for real-time small object detection, advancing both the theoretical understanding and practical deployment of vision systems in challenging visual environments.

## Method

**Fig. 1:**
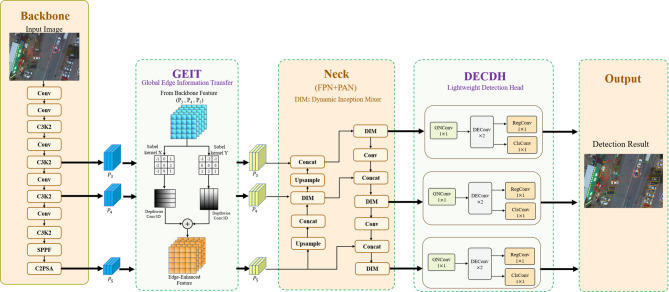
GDD-YOLO overall architecture overview.

This paper selects YOLOv11s as the baseline model due to its fewer parameters and faster response time, and proposes a Global Edge Information Transfer (GEIT) module and a Dynamic Inception Mixer (DIM) module. The GEIT module propagates the edge information extracted from shallow features throughout the backbone network and integrates it with features at different scales. The DIM module enhances the model’s adaptability and flexibility by dynamically adjusting convolutional kernel parameters to capture multi-scale target information. Furthermore, to meet the demands of real-time small object detection, we design a lightweight detection head (DECDH) that employs a shared convolution strategy aimed at reducing both the computational load and parameter count of the detection head. The overall architecture of GDD-YOLO is illustrated in Fig. 1.

**Table 1:** Summary of the main notations, abbreviations in this paper.

Symbol	Meaning
$\:X$	Input feature map
$\:E$	Edge feature/edge map
$\:{E}_{s}$	Edge feature at scale $\:s$
$\:F$	Feature map
$\:{F}_{in}$	Input feature map to a module
$\:{F}_{out}$	Output feature map of a module
$\:{F}_{s}$	Feature map at scale $\:s$
$\:{G}_{x}$	Horizontal Sobel gradient kernel
$\:{G}_{y}$	Vertical Sobel gradient kernel
$\:s$	Scale index in the GEIT/MSEIG module
$\:N$	Number of scales
$\:C$	Number of channels
$\:c$	Channel index
$\:H$	Height of the convolution kernel/feature map, depending on context
$\:W$	Width of the convolution kernel/feature map, depending on context
(*x*,* y*)	Spatial position in a feature map
$\:{K}_{d}$	Dynamic kernel weight
$\:{K}_{c}$	Dynamic kernel weight of the $\:c$-th channel
$\:{Y}_{c}$	Activation response/output feature of the $\:c$-th channel
$\:\odot\:$	Element-wise multiplication
$\:\oplus\:$	Element-wise summation/residual summation
	Feature concatenation
$\:\sigma\:$	Activation function

### Global edge information transfer (GEIT) module

Accurate edge perception is crucial for object detection tasks, particularly for the localization of small objects and boundaries. Existing methods typically rely on standard convolutional layers, which may suppress subtle edge cues. To address this limitation, we propose a novel Global Edge Information Transfer (GEIT) module that explicitly integrates Sobel-based edge extraction. By fusing edge information across multiple scales, this module enhances the feature representations of the backbone network, significantly improving the model’s ability to discern object boundaries in complex scenes.

**Fig. 2:**
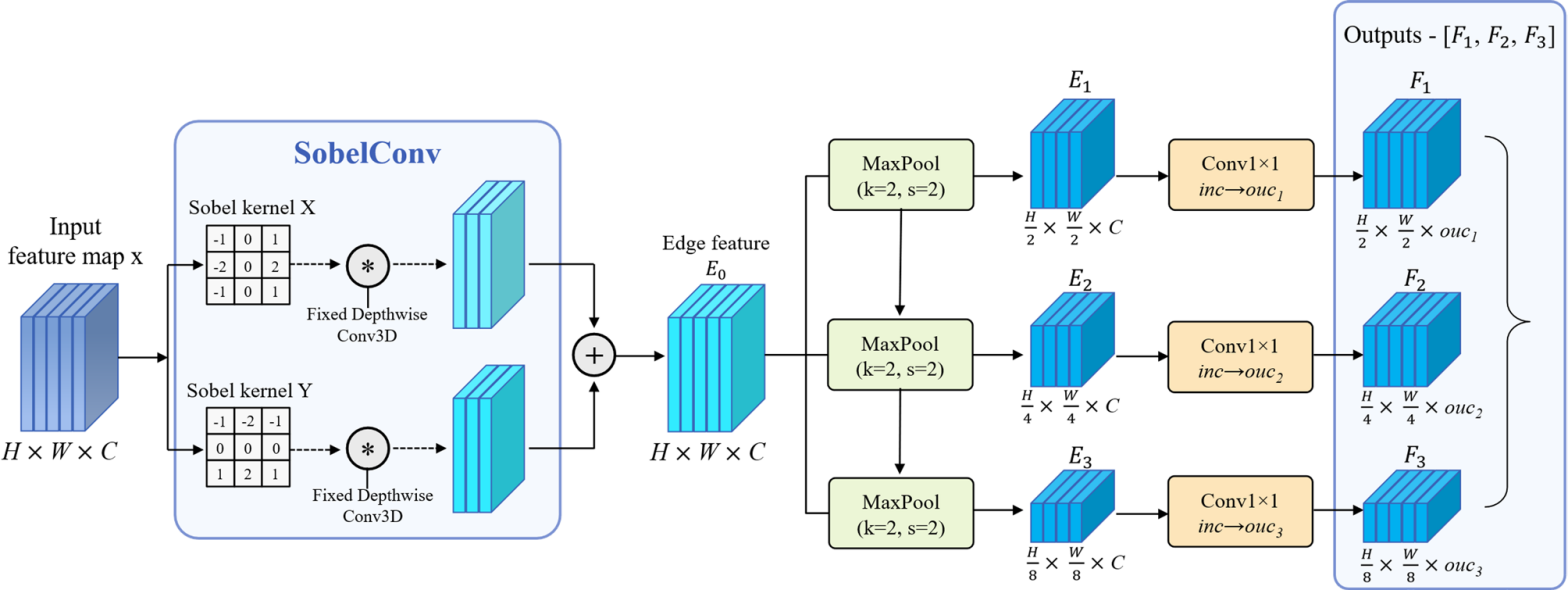
The structure of MSEIG within the GEIT module.

Within GEIT, we design a Multiscale Edge Information Generator (MSEIG). This sub-module employs fixed Sobel operators to preserve high-frequency details. The structure of MSEIG within the GEIT module is illustrated in Fig. 2.

Given an input feature map $\:{X}\in{{R}}^{{B\times{C}\times{H}\times{W}}}$, the horizontal and vertical gradient kernels are defined as follows:

1 $$\:{\mathrm{K}}_{\mathrm{x}}\mathrm{=}\left[\begin{array}{ccc}\mathrm{1}&\:\mathrm{2}&\:\mathrm{1}\\\:\mathrm{0}&\:\mathrm{0}&\:\mathrm{0}\\\:\mathrm{-1}&\:\mathrm{-2}&\:\mathrm{-1}\end{array}\right]\mathrm{,}\text{}{\mathrm{K}}_{\mathrm{y}}\mathrm{=}{\mathrm{K}}_{\mathrm{x}}^{\mathrm{T}}$$

These kernels are expanded to C channels via grouped 3D convolution, implemented through the 3D formulation of depthwise convolution:

2 $$\:{\mathrm{M}}_{\mathrm{base}}\mathrm{=}\underset{\mathrm{Horizontal\:response}}{\underbrace{\mathrm{Conv3D}\left(\mathrm{X;}{\mathrm{K}}_{\mathrm{x}}\right)}}\mathrm{+}\underset{\mathrm{Vertical\:response}}{\underbrace{\mathrm{Conv3D}\left(\mathrm{X;}{\mathrm{K}}_{\mathrm{y}}\right)}}$$

The Sobel-based gradient extraction itself introduces no additional learnable parameters and only limited computational overhead and avoids gradient noise interference during training. To capture edge semantics at varying granularities, we first generate the base edge map, then apply cascaded max pooling for downsampling. Finally, each scaled feature is processed by channel-specific 1 × 1 convolutions:

3 $$\:{E}_{0}=SobelConv\left(X\right)\in\:{\mathbb{R}}^{B\times\:{C}_{in}\times\:H\times\:W}$$

4 $$\:{E}_{s}={MaxPool}_{2\times\:2}^{\left(s\right)}\left({E}_{0}\right)\in\:{\mathbb{R}}^{B\times\:{C}_{in}\times\:\frac{H}{{2}^{s}}\times\:\frac{W}{{2}^{s}}}$$

5 $$\:{F}_{s}={Conv}_{1\times\:1}^{\left(s\right)}\left({E}_{s}\right)\in\:{\mathbb{R}}^{B\times\:{ouc}_{s}\times\:\frac{H}{{2}^{s}}\times\:\frac{W}{{2}^{s}}}$$

*Where*:

*s - the scale index*,

*s = 1*,*2*,* …*,*N (N = len(oucs))*,

$\:{\:\:\:\:ouc}_{s}$ *- the output channel dimension for scale.*

Regarding downsampling strategy, we balance feature preservation against resolution compression. MaxPooling outperforms Average Pooling (AvgPooling) for retaining and enhancing edge information while downsampling. Its local maxima extraction mechanism effectively preserves high-frequency edge responses and suppresses interference from smooth regions, better reflecting edge structures. Conversely, AvgPooling’s local averaging property, while beneficial for feature smoothing and noise suppression, dilutes gradient transition responses. This significantly reduces edge sharpness and underperforms MaxPooling in preserving fine details and edge information.

**Fig. 3:**
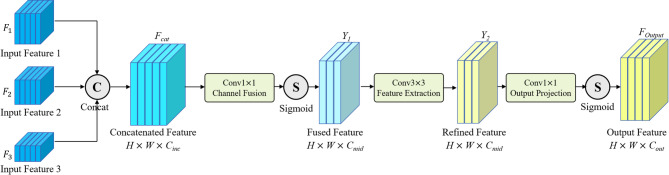
The structure of ConvEdgeFusion in the GEIT module.

For feature fusion within GEIT, ConvEdgeFusion intelligently integrates edge information with standard convolutional features through a novel cross-channel fusion approach. The structure of ConvEdgeFusion in the GEIT module is illustrated in Fig. 3.

First, channel fusion convolution performs cross-channel fusion of edge information and standard convolutional features, enabling the model to better integrate features from different sources. Subsequently, a 3 × 3 feature extraction convolution further processes the fused features to enhance the model’s capability to capture local details. Finally, a 1 × 1 convolution adjusts the output feature dimensionality. This process is formulated as:

6 $$\:{\mathrm{F}}_{\mathrm{cat}}\mathrm{=}\mathrm{Concat}\left({\mathrm{F}}_{\mathrm{1}}\mathrm{,}{\mathrm{F}}_{\mathrm{2}}{,\dots,}{\mathrm{F}}_{\mathrm{K}}\right)\in{\mathrm{R}}^{{B\times}\left(\sum\:_{\mathrm{k=1}}^{\mathrm{K}}{\hspace{0.05em}}{\mathrm{C}}_{\mathrm{k}}\right){\times{H}\times{W}}}$$

7 $$\:{\mathrm{Y}}_{\mathrm{1}}{=\sigma}\left({\mathrm{Conv}}_{{1\times1}}\left({\mathrm{F}}_{\mathrm{cat}}\right)\right)\in{\mathrm{R}}^{{B\times}{\mathrm{C}}_{\mathrm{mid}}{\times{H}\times{W}}}$$

8 $$\:{\mathrm{Y}}_{\mathrm{2}}{=\sigma}\left({\mathrm{Conv}}_{{3\times3}}\left({\mathrm{Y}}_{\mathrm{1}}\right)\right)\in{\mathrm{R}}^{{B\times}{\mathrm{C}}_{\mathrm{mid}}{\times{H}\times{W}}}$$

9 $$\:{\mathrm{F}}_{\mathrm{Output}}\mathrm{=}{\mathrm{Conv}}_{{1\times1}}\left({\mathrm{Y}}_{\mathrm{2}}\right)\in{\mathrm{R}}^{{B\times}{\mathrm{C}}_{\mathrm{out}}{\times{H}\times{W}}}$$

*Where*:

$$\:{\mathrm{C}}_{\mathrm{mid}}\mathrm{=}{\mathrm{C}}_{\mathrm{out}}/\mathrm{2}$$

σ - *the activation function*,* and all convolutional layers incorporate BatchNormalization(BN) and activation functions.*

The GEIT module extracts critical edge features from shallow layers, generates multi-scale edge information feature maps, and propagates them to the corresponding scales throughout the backbone for fusion. This process achieves cross-scale edge information enhancement and significantly improves the localization accuracy of small objects.

The main purpose of GEIT is to compensate for the structural information loss caused by repeated downsampling in the backbone. Since small objects occupy only a limited number of pixels, their texture cues are often weak, whereas boundary responses remain relatively informative for localization. GEIT therefore extracts edge features from high-resolution shallow maps using fixed Sobel operators, progressively constructs aligned edge representations at multiple scales via max pooling, and injects them into deeper backbone stages through ConvEdgeFusion. In this way, GEIT supplies explicit boundary priors to multi-scale semantic features and improves the network’s sensitivity to object contours under cluttered backgrounds. The ablation results in Tables 1, 2, 3 and 4 verify this effect: compared with YOLOv11-S, adding GEIT improves AP from 23.8 to 24.7 and AP50 from 39.2 to 40.7. These results show that edge-aware feature transfer is beneficial for small-object localization.

### Dynamic inception mixer (DIM)

**Fig. 4:**
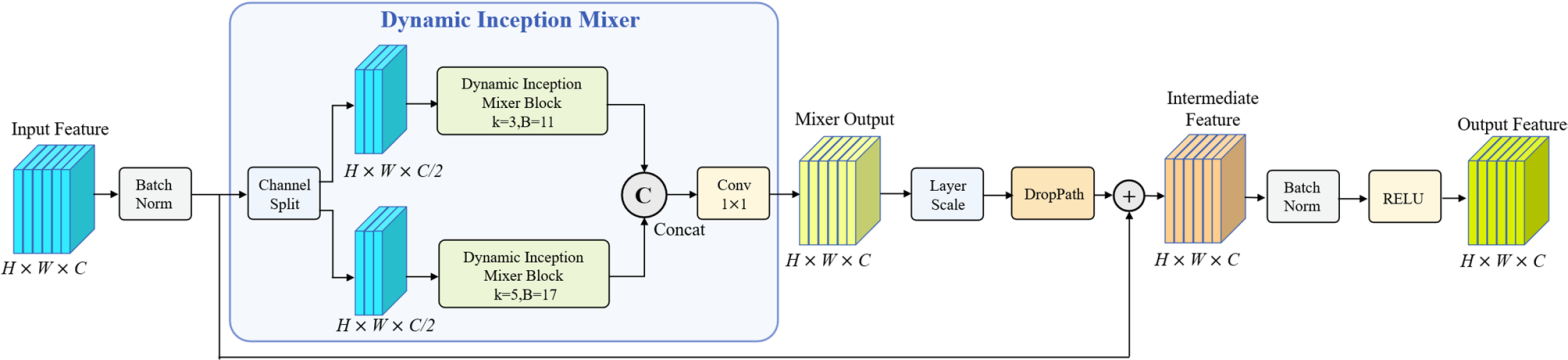
Overall structure of DIM.

In the dynamic hybrid convolution network, the DIM Block introduces a Dynamic Kernel Weight(DWK) mechanism. This mechanism assigns adaptive weights to each convolutional kernel, enabling the network to dynamically adjust kernel usage based on the input feature map’s requirements. The overall structure of DIM is illustrated in Fig. 4.

This approach breaks the static parameter constraints of traditional fixed convolutional kernels. During forward propagation, it reconstructs kernel parameters in real-time according to the local response characteristics of input features. This input-adaptive adjustment effectively captures multi-scale and diverse feature information.

Dynamic Weight Generation: Global average pooling is applied to obtain channel-wise global information. Dynamic kernel weights $\:{{\omega}}_{\mathrm{c}}\in{\text{}\mathrm{R}}^{{C\times1\times1}}$ are generated via a Softmax layer:

10 $$\:{{\omega}}_{\mathrm{c}}\mathrm{=}\mathrm{Softmax}\left({\mathrm{Conv}}_{{1\times1}}\mathrm{(}\frac{\mathrm{1}}{{H\times{W}}}\sum\:_{\mathrm{i}\mathrm{=0}}^{\mathrm{H}}{\hspace{0.05em}}\sum\:_{\mathrm{j=0}}^{\mathrm{W}}{\hspace{0.05em}}{\mathrm{X}}^{\mathrm{D}}\left(\mathrm{i,j}\right)\mathrm{)}\right)$$

The dynamic hybrid convolution network employs depthwise convolution, which significantly reduces both parameter count and computational complexity while maintaining detailed processing of input feature maps. Through dynamic kernel adjustment, this approach not only enhances model performance on diverse inputs but also reduces computational redundancy. After obtaining the dynamic kernel weights $\:{{\omega}}_{\mathrm{c}}$, depthwise convolution yields the output feature map $\:{\mathrm{Y}}_{\mathrm{c}}$. This operation performs spatially independent convolution per input channel, achieving channel decoupling, formulated as:

11 $$\:{\mathrm{Y}}_{\mathrm{c}}\left(\mathrm{i,j}\right)\mathrm{=}{\sum\:}_{{\mathrm{k}}_{\mathrm{x}}\mathrm{=-}\lfloor\mathrm{K}/\mathrm{2}\rfloor}^{\lfloor\mathrm{K}/\mathrm{2}\rfloor}{\sum\:}_{{\mathrm{k}}_{\mathrm{y}}\mathrm{=-}\lfloor\mathrm{K}/\mathrm{2}\rfloor}^{\lfloor\mathrm{K}/\mathrm{2}\rfloor}{{\omega}}_{\mathrm{c}}\left({\mathrm{k}}_{\mathrm{x}}\mathrm{,}{\mathrm{k}}_{\mathrm{y}}\right)\odot{\mathrm{X}}^{\mathrm{D}}\left(\mathrm{i,j}\right)$$

*Where*:

$\:{\mathrm{X}}^{\mathrm{D}}\in\text{}{\text{}\mathrm{R}}^{{H\times{W}}}$ *- Feature map of the c-th input channel*.

$\:{{\omega}}_{\mathrm{c}}\in\text{}{\mathrm{R}}^{{C\times1\times1}}$ *- Dynamic kernel weight for channel c*.

$\:{\mathrm{Y}}_{\mathrm{c}}\in{\text{}\mathrm{R}}^{{\mathrm{H}}^{{\prime\:}}{\times}{\mathrm{W}}^{{\prime\:}}}$ *- Activation response of the c-th output channel*.

$\odot$ *- Element-wise multiplication*.

$\:\left(\mathrm{i}\mathrm{,}\text{}\mathrm{j}\right)$ *- Spatial position in output feature map*.

$\:{k}_{x}$, $\:{k}_{y}$ *- Convolution kernel height and width*.

**Fig. 5:**
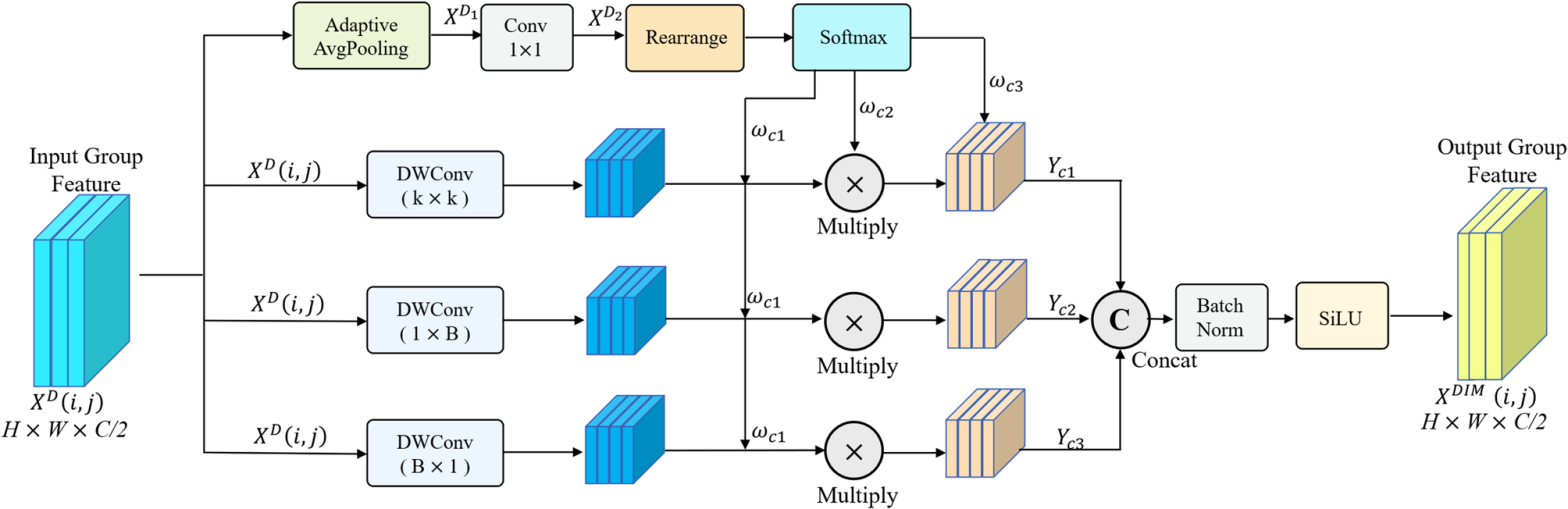
DIM Block structure.

The Dynamic Inception Mixer (DIM) extracts and adaptively aggregates information across multiple scales through multi-scale convolutional kernel fusion. This cross-scale feature extraction enables the network to capture fine-grained details at various resolutions, overcoming reliance on any single fixed-size kernel. Consequently, it substantially enhances the model’s representation capacity for complex scenes. Simultaneously, a residual summation integrates the convolved feature maps with the input features. This preserves effective propagation of both low-level spatial details and high-level semantic information throughout the network. Such design not only facilitates full utilization of multi-level features but also mitigates gradient vanishing and feature degradation issues inherent in deep architectures. The DIM Block structure is shown in Fig. 5.

Following depthwise convolution yielding feature map $\:{\mathrm{Y}}_{\mathrm{c}}$, the outputs are separately mapped into three feature components $\:{\mathrm{Y}}_{\mathrm{c1}}$, $\:{\mathrm{Y}}_{\mathrm{c2}}$, and $\:{\mathrm{Y}}_{\mathrm{c3}}$. These branch features are then concatenated to form $\:{\mathrm{X}}^{\mathrm{DIM}}$, which integrates multi-level information:

12 $$\:{\mathrm{X}}^{\mathrm{DIM}}\mathrm{=}\left({{\mathrm{Y}}_{\mathrm{c1}}}^{\text{}}\right)\oplus\left({{\mathrm{Y}}_{\mathrm{c2}}}^{\text{}}\right)\oplus\left({{\mathrm{Y}}_{\mathrm{c3}}}^{\text{}}\right)$$

Through dynamic kernel selection, cross-layer feature fusion, and computationally efficient architecture, the Dynamic Inception Mixer (DIM) effectively addresses three key limitations of traditional convolutional neural networks: (1) inadequate multi-scale feature extraction, (2) suboptimal information integration, and (3) computational inefficiency. Our design not only enhances the network’s feature adaptability and architectural flexibility but also demonstrates superior operational robustness and computational efficiency in practical applications.

The purpose of DIM is to improve scale adaptability without introducing heavy computational overhead. Conventional static convolutions use a fixed receptive field for all inputs, which is suboptimal for VisDrone scenes where object sizes vary considerably because of altitude, viewpoint, and density differences. DIM addresses this limitation by dynamically aggregating multiple lightweight convolution branches with different receptive fields according to the current input response. As a result, the block can emphasize more suitable spatial contexts for different feature patterns instead of relying on a single fixed kernel. This design enhances multi-scale representation and feature flexibility while remaining efficient. As shown in Tables 5 and 6, after GEIT is introduced, adding DIM further improves AP from 24.7 to 26.2 and AP50 from 40.7 to 42.0, while reducing parameters from 11.2 M to 9.3 M and FLOPs from 23.4G to 22.3G. This demonstrates that DIM not only strengthens feature extraction for small objects, but also improves the overall accuracy-efficiency trade-off.

### Lightweight shared detail-enhanced convolution detection head (DECDH)

The DECDH processes multi-scale features using Group Normalization Convolution (GNConv) followed by two consecutive Detail-Enhanced Convolution (DEConv) layers. This design preserves critical spatial details while achieving cross-scale feature consistency, where we further employ a shared convolution strategy to substantially reduce parameter count. Processed features are then split into parallel branches for bounding box regression and classification, with learnable scale parameters dynamically adjusting regression outputs per feature level. The DECDH architecture is illustrated in Fig. 1.

Detail-Enhanced Convolution (DEConv) integrates a 3 × 3 group convolution branch for capturing localized details and a 5 × 5 depthwise convolution branch for extracting broader contextual features, with element-wise summation through residual connections to amplify high-frequency details, formalized as:

13 $$\:\text{}\mathrm{DEConv}\left(\mathrm{X}\right)\sigma\left(\mathrm{GN}\left({\mathrm{Conv}}_{{3\times3}}^{\mathrm{group}}\left(\mathrm{X}\right)\right)\mathrm{+}{\mathrm{Conv}}_{{5\times5}}^{\mathrm{dw}}\left(\mathrm{X}\right)\right)$$

*Where*:

$\sigma$ *denotes the ReLU activation function and GN represents*.

*Group Normalization.*

Conventional detection heads process each feature scale independently, leading to parameter redundancy and inconsistent feature representations. Our shared convolution approach effectively addresses these limitations, formulated as:

14 $$\:{\mathrm{F}}_{\mathrm{i}}^{\mathrm{out}}\mathrm{=}{\mathrm{DEConv}}_{\mathrm{2}}\left({\mathrm{DEConv}}_{\mathrm{1}}\left({\mathrm{Conv}}_{\mathrm{i}}^{{1\times1}}\left({\mathrm{F}}_{\mathrm{i}}^{\mathrm{in}}\right)\right)\right)$$

*Where*:

*i denotes the feature level index (P3-P5).*

The lightweight DECDH introduces Detail-Convolution (DEConv) to enhance feature representation and improve the model’s generalization capability. Meanwhile, its shared convolution strategy reduces both the computational cost and the number of parameters in the detection head, thereby better satisfying the requirements of real-time small object detection.

The detection head directly affects classification and localization performance, yet conventional per-scale heads contain substantial parameter redundancy. A purely lightweight replacement may reduce this cost but often weakens detail representation, which is unfavorable for small-object detection. DECDH is designed to balance efficiency and detail preservation. Specifically, shared convolutions are used across feature levels to reduce repeated parameters, while DEConv combines local detail extraction and wider contextual perception to maintain discriminative representation for small targets. In addition, the decoupled prediction branches and learnable scale factors preserve regression flexibility across pyramid levels. The results in Table 6 show that replacing the original head with DECDH keeps AP at 26.2 and slightly improves AP50 from 42.0 to 42.1, while further reducing parameters from 9.3 M to 7.9 M and FLOPs from 22.3G to 19.3G. This confirms that DECDH improves deployment efficiency without sacrificing detection accuracy.

## Experiments

### Dataset and experimental setup

**Fig. 6:**
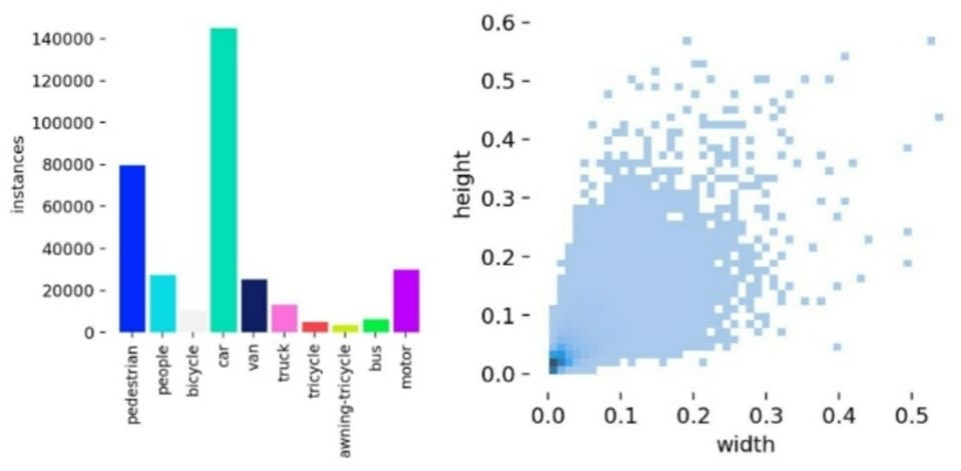
Category-wise object statistics.

Extensive experiments were conducted on the VisDrone2019 small object dataset, developed by Tianjin University researchers, which comprises 10,209 images captured by diverse drone-mounted cameras across varied scenarios under different weather and illumination conditions. The dataset is partitioned into 6,471 training images, 548 validation images, and 1,580 test images, featuring 10 object categories characterized by a high proportion of small objects, dense target distribution, and multi-category complexity. Category-wise object statistics are shown in Fig. 6, while experimental environment configurations are detailed in Table 2.

**Table 2:** Configuration table for the experimental environment.

Name	Model
System	Ubuntu 20.04
Programming language	Python 3.10.1
GPU	NVIDIA GeForce RTX3090
GPU memory	24GB
Epochs	300
Batch size	8
Learning rate	0.01
Optimizer	SGD
Momentum	0.937

### Model performance evaluation metrics

This study employs three key metrics: mean Average Precision (mAP) to evaluate overall detection and classification accuracy; Precision (P), measuring the proportion of correctly identified positive instances among all predicted positives; and Recall (R), reflecting the proportion of actual positives correctly identified. The computational expressions for Recall (Eq. 16), Precision (Eq. 15), and mean Average Precision (Eq. 18) are defined as follows:

15 $$\:\mathrm{Precision=}\frac{\mathrm{TP}}{\mathrm{TP+FP}}{\times100\%}$$

16 $$\:\mathrm{Recall=}\frac{\mathrm{TP}}{\mathrm{TP+FN}}{\times100\%}$$

17 $$\:\mathrm{AP=}{\int\:}_{\mathrm{0}}^{\mathrm{1}}{\hspace{0.05em}}\mathrm{P}\left(\mathrm{R}\right)\mathrm{dR}$$

18 $$\:\mathrm{mAP}\mathrm{=}\frac{\sum\:_{\mathrm{i}\mathrm{=1}}^{\mathrm{K}}{\hspace{0.05em}}\mathrm{A}{\mathrm{P}}_{\mathrm{i}}}{\mathrm{K}}{\times100\%}$$

True Positives (TP) denote the number of correctly predicted positive samples; False Positives (FP) represent negative samples incorrectly predicted as positive; False Negatives (FN) indicate positive samples erroneously predicted as negative, where K denotes the total number of target categories. To comprehensively evaluate computational efficiency, additional metrics are introduced: total parameters, model size, GFLOPs (giga floating-point operations), and FPS (frames per second).

### Comparative analysis of state-of-the-art algorithms

**Fig. 7:**
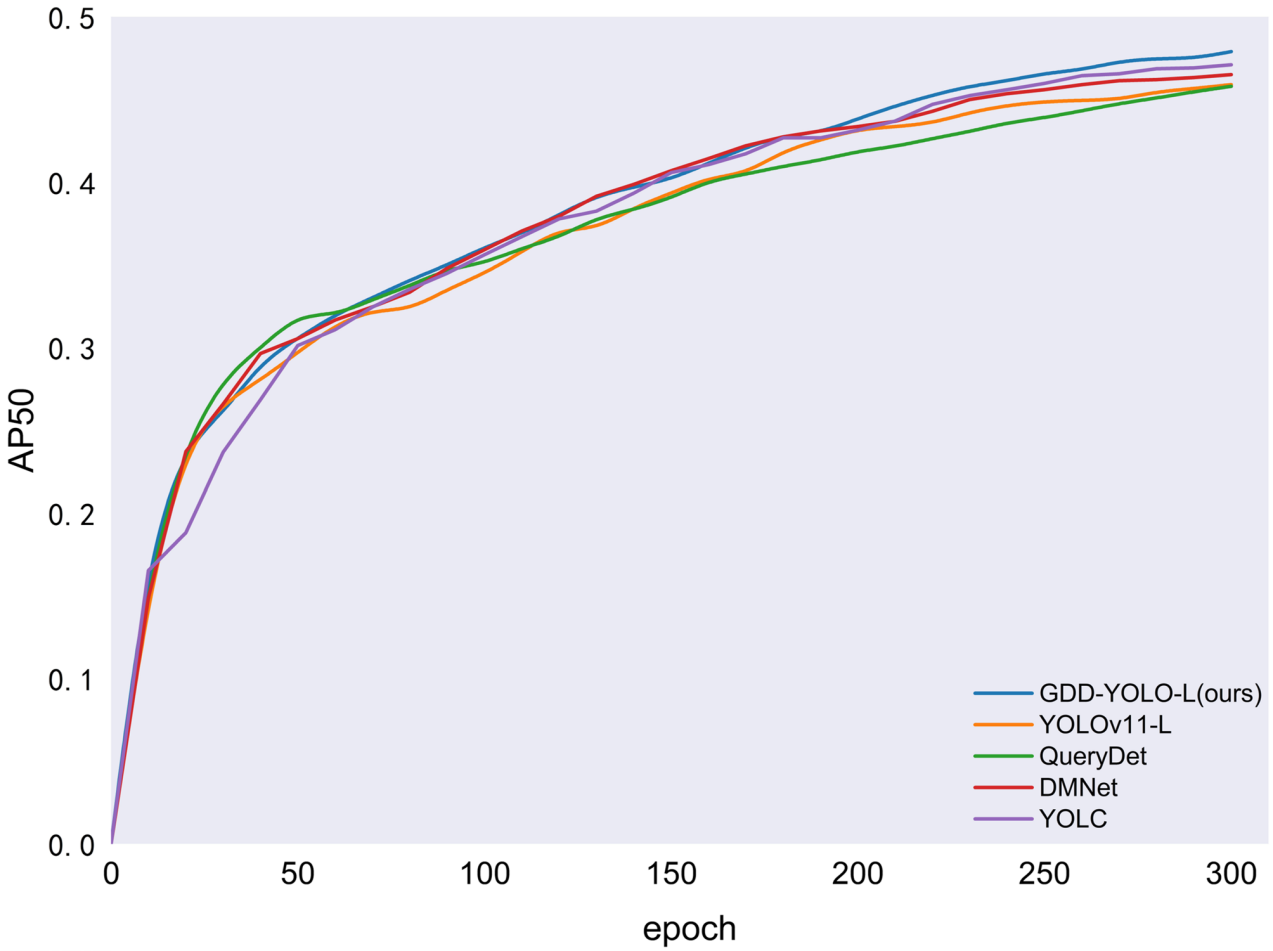
Comparative AP50 curves across evaluated models.

**Fig. 8:**
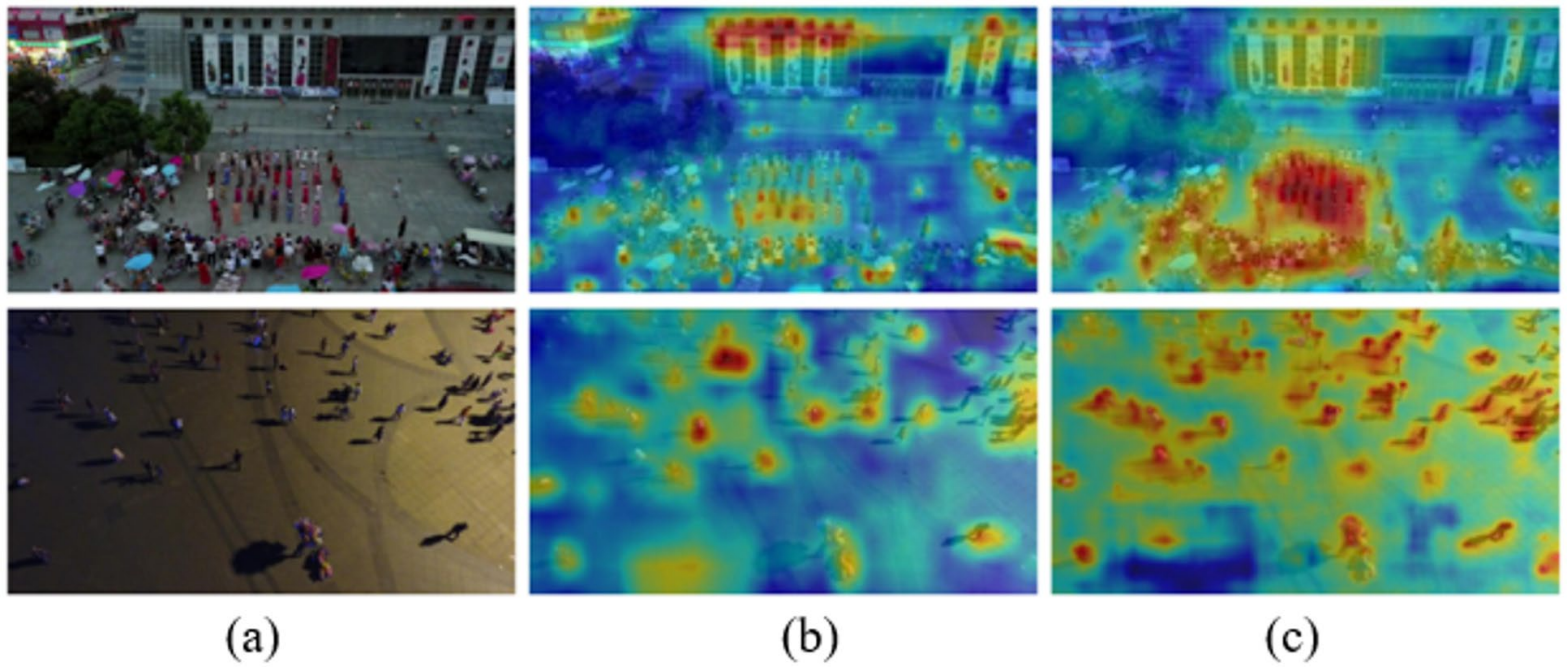
Feature activation heatmaps of baseline model versus proposed method (GDD-YOLO). (**a**) Original Image (**b**) YOLOv11-s (**c**) GDD-YOLO (Ours).

Comparative evaluation against leading real-time detectors across model scales on VisDrone, with AP50 curves contrasted in Figs. 7 and 8. Table 3 presents a controlled comparison on the VisDrone dataset under a unified experimental setting. The results show that GDD-YOLO consistently achieves a more favorable accuracy-efficiency trade-off than the baseline real-time detectors. Specifically, compared with YOLOv11-S, GDD-YOLO-S improves AP from 23.8 to 26.2 and AP50 from 39.2 to 42.1, while reducing the number of parameters from 9.5 M to 7.9 M and FLOPs from 22.6G to 19.3G, and slightly increasing FPS from 135 to 139. Similarly, compared with RT-DETR-R34 and RT-DETR-R50, GDD-YOLO-M/L achieve higher AP (+ 1.3/+0.8), substantially fewer parameters, lower computational cost, and faster inference, although their AP50 values are slightly lower by 0.2 and 0.4 points, respectively. Since AP is averaged over multiple IoU thresholds and therefore reflects overall localization quality more strictly than AP50 alone, these results indicate that GDD-YOLO provides a better overall balance between detection accuracy and efficiency for real-time small object detection.

**Table 3:** Comparison of AP (%), parameters, and frame rates between our method and different real-time object detectors on the VisDrone test dataset.

Model	AP	$\:\mathrm{A}{\mathrm{P}}_{\mathrm{50}}$	FLOPs	Params	FPS
YOLOv8-N	19.2	32.7	8.9 G	3.4 M	171
YOLOv8-S	23.4	38.6	28.6 G	12.2 M	129
YOLOv8-M	26.8	43.2	78.9 G	26.9 M	81
YOLOv8-L	28.3	45.5	167.2 G	43.8 M	59
YOLOv11-N	19.8	33.1	6.8G	2.7 M	179
YOLOv11-S	23.8	39.2	22.6 G	9.5 M	135
YOLOv11-M	27.1	43.6	71.3G	22.6 M	87
YOLOv11-L	28.6	45.9	120.3 G	28.4 M	62
RT-DETR-R34	27.2	46.0	96.0 G	33.0 M	86
RT-DETR-R50	28.8	48.3	138.5 G	43.2 M	63
GDD-YOLO-N	21.1	34.8	6.0 G	2.5 M	182
GDD-YOLO-S	26.2	42.1	19.3 G	7.9 M	139
GDD-YOLO-M	28.5	45.8	57.9 G	18.5 M	89
GDD-YOLO-L	29.6	47.9	99.4 G	22.8 M	68

Concurrently, for resource-constrained platforms, Table 4 presents comparative evaluation results of different-scale GDD-YOLO models against other state-of-the-art real-time detectors. Table 4 summarizes the results reported in previous studies on the VisDrone dataset and is included to position the proposed method with respect to the broader literature. Because these methods may differ in input resolution, training schedule, data augmentation, and implementation details, this comparison should be interpreted as a reference-level comparison rather than a strictly controlled head-to-head evaluation. Within this reference set, GDD-YOLO achieves the best reported AP/AP50/AP75 values (29.6/47.9/31.7), which demonstrates that the proposed method remains highly competitive among recent small object detectors.

**Table 4:** Comparative AP (%) performance versus state-of-the-art detectors on the VisDrone test dataset.

Model	AP	$\:\mathrm{A}{\mathrm{P}}_{\mathrm{50}}$	$\:\mathrm{A}{\mathrm{P}}_{\mathrm{75}}$
TPH-YOLOv5	27.1	46.3	28.4
CEASC^24^	27.3	46.7	29.1
QueryDet^25^	27.6	45.8	28.8
DMNet^26^	28.5	46.5	30.4
YOLC^27^	28.9	47.1	28.5
Efficient YOLOv8^28^	28.6	46.9	30.2
ARGT^29^	29.0	47.3	30.9
HE-YOLOv9^30^	28.8	47.1	31.1
LGCA-Transformer^31^	29.4	47.5	31.5
Efficient YOLOv12^32^	29.2	47.6	31.7
GDD-YOLO(ours)	29.6	47.9	31.7

**Fig. 9:**
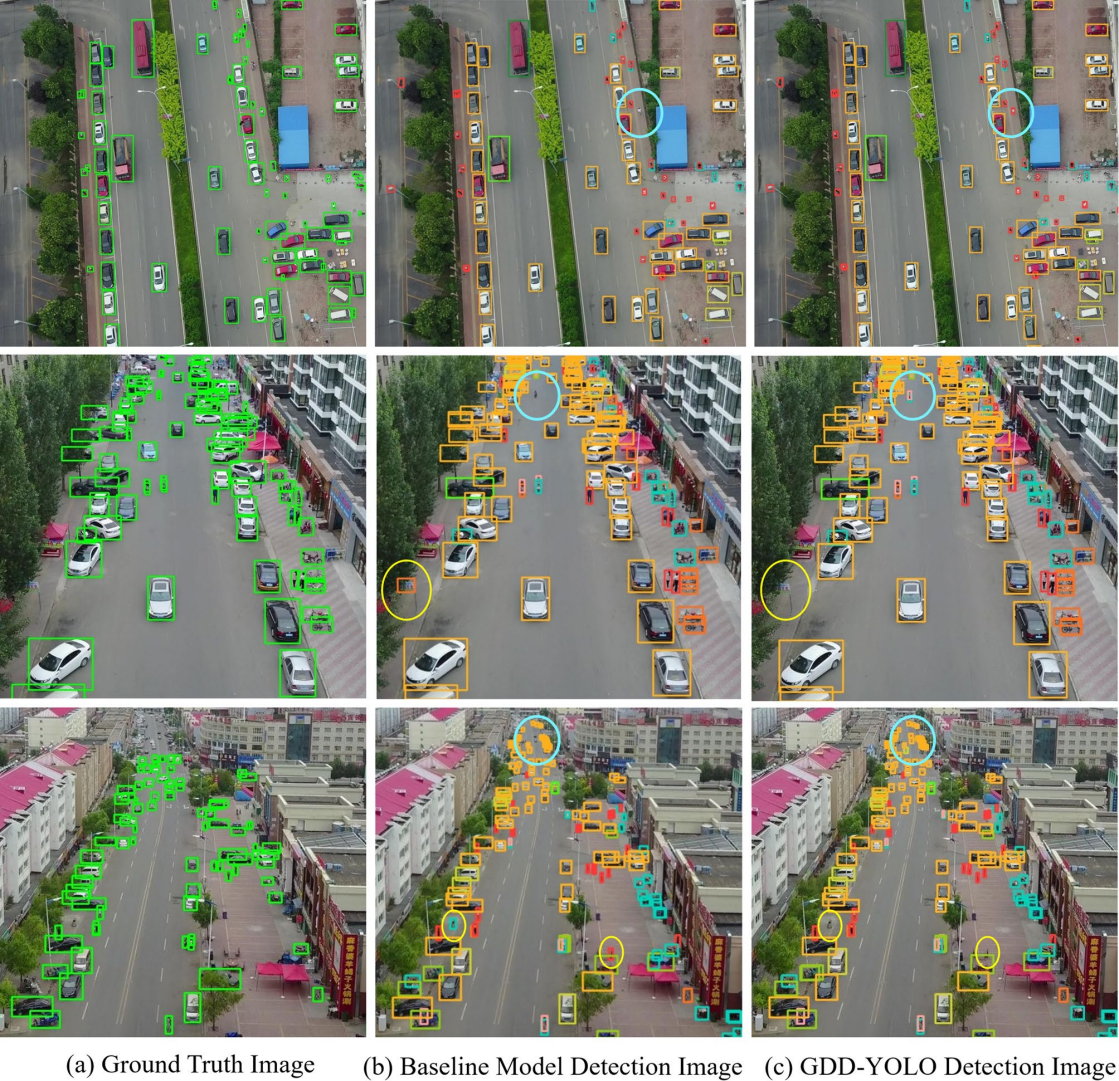
Detection comparison between YOLOv11-s and GDD-YOLO, where light blue circles denote missed detections by YOLOv11-s and yellow circles indicate its false detections.

These results robustly validate the effectiveness and superiority of the GDD-YOLO framework. Our method focuses on optimizing object representation through enhanced perception of subtle edge features and improved multi-scale feature fusion capabilities. As evidenced by qualitative evaluations in Fig. 9, the proposed approach significantly boosts detection performance under cluttered background interference.

**Fig. 10:**
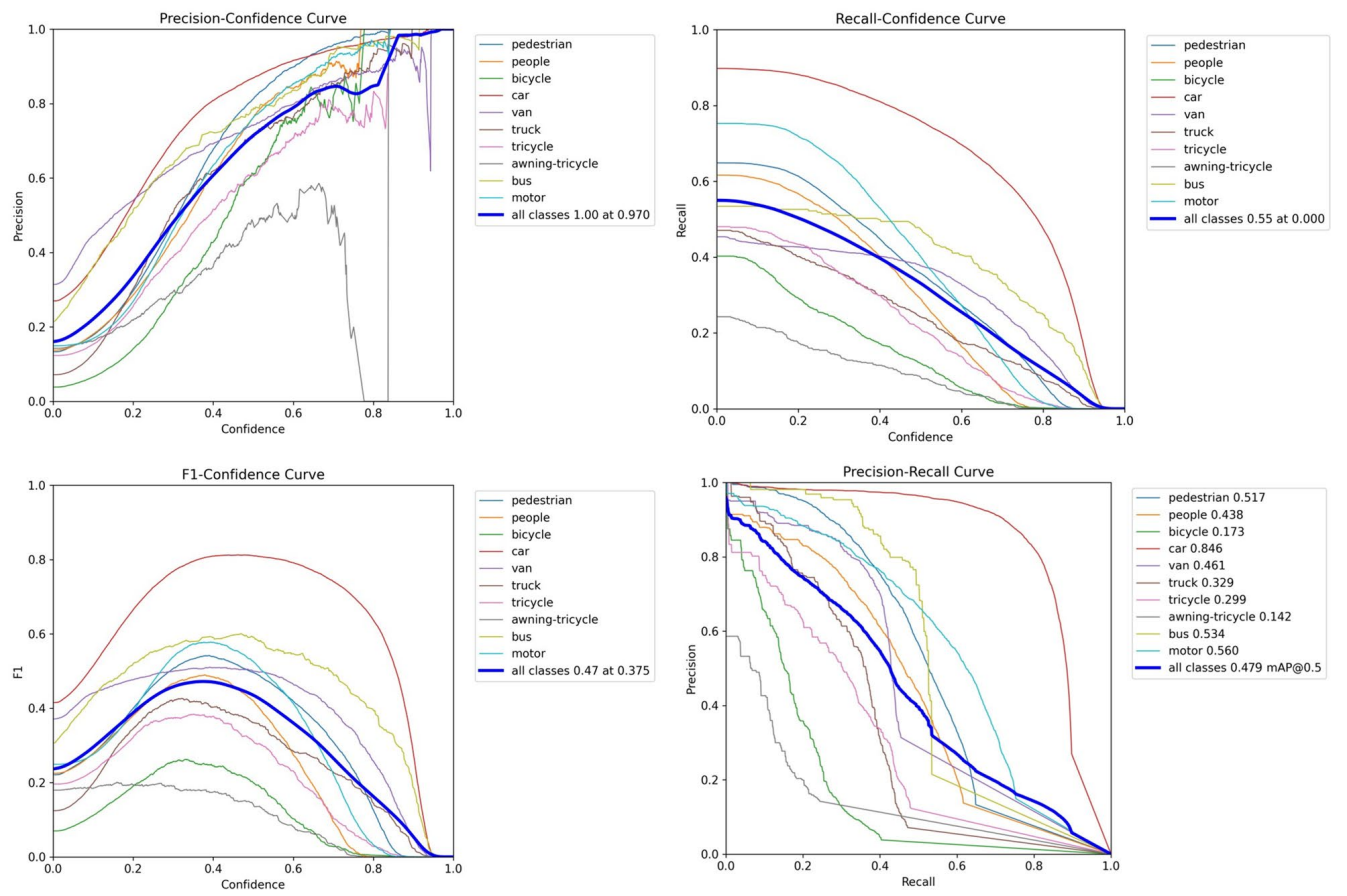
Class-wise performance curves of GDD-YOLO on the VisDrone dataset. Different colored curves represent different object classes, and the bold blue curve denotes the overall performance across all classes. In (d), the value for each class indicates its AP, while the overall result is reported as mAP@0.5.

These results robustly validate the effectiveness and superiority of the GDD-YOLO framework. Our method focuses on optimizing object representation through enhanced perception of subtle edge features and improved multi-scale feature fusion capabilities. As evidenced by qualitative evaluations in Figs. 9 and 10 the proposed approach significantly boosts detection performance under cluttered background interference.

**Table 5:** Comparison of deployment-oriented computational efficiency and resource usage of different methods on the VisDrone test dataset.

Model	Latency (ms/image)	FPS	Model size (MB)	Peak GPU memory(GB)
YOLOv8-N	5.85	171	13.6	1.28
YOLOv8-S	7.75	129	48.8	1.68
YOLOv8-M	12.35	81	107.6	2.35
YOLOv8-L	16.95	59	175.2	3.30
YOLOv11-N	5.59	179	10.8	1.22
YOLOv11-S	7.41	135	38.0	1.52
YOLOv11-M	11.49	87	90.4	2.15
YOLOv11-L	16.13	62	113.6	2.65
RT-DETR-R34	11.63	86	132.0	2.55
RT-DETR-R50	15.87	63	172.8	3.05
GDD-YOLO-N	5.49	182	10.0	1.20
GDD-YOLO-S	7.19	139	31.6	1.42
GDD-YOLO-M	11.24	89	74.0	1.95
GDD-YOLO-L	14.71	68	91.2	2.35

In practical applications, hardware deployment should be assessed not only by detection accuracy but also by storage cost, runtime memory footprint, and inference efficiency. Therefore, we further analyze the deployment-related resource characteristics of GDD-YOLO. As shown in Table 5, GDD-YOLO-S achieves a latency of 7.19 ms/image and 139 FPS, while its model size and peak GPU memory are 31.6 MB and 1.42 GB, respectively. Compared with YOLOv11-S, this corresponds to a reduction of 6.4 MB in model storage and 0.10 GB in peak runtime memory, together with an AP improvement from 23.8 to 26.2. In addition, the lower parameter count (7.9 M) and FLOPs (19.3 G) reported in Table 3 further demonstrate that the proposed model is more suitable for resource-constrained deployment scenarios. To avoid overstatement, we clarify that the current measurements were obtained under a unified RTX3090 environment; therefore, they should be interpreted as deployment-oriented resource indicators rather than hardware-specific embedded benchmarks. Actual validation on dedicated edge hardware will be investigated in future work.

### Ablation study and data analysis

This study evaluates the impact of GDD-YOLO’s core modules on final detection performance through ablation experiments conducted on the VisDrone dataset. All ablation studies use YOLOv11-S as the baseline model. Results are presented in Table 6.

**Table 6:** Module ablation study on GEIT, DIM, DECDH in VisDrone dataset.

GEIT	DIM	DECDH	AP	$\:\mathrm{A}{\mathrm{P}}_{\mathrm{50}}$	Params	FLOPs
×	×	×	23.8	39.2	9.5 M	22.6 G
✓	×	×	24.7	40.7	11.2 M	23.4 G
✓	✓	×	26.2	42.0	9.3 M	22.3 G
✓	✓	✓	26.2	42.1	7.9 M	19.3G

Ablation results in Table 6 demonstrate that each proposed module significantly enhances YOLOv11’s detection performance on the VisDrone dataset. To more clearly interpret the role of each module, Table 6 should be read progressively. GEIT primarily improves boundary-aware localization, yielding + 0.9 AP and + 1.5 AP50 over the baseline. DIM further enhances scale-adaptive feature extraction and contributes an additional + 1.5 AP and + 1.3 AP50 over the GEIT-enhanced model, while simultaneously reducing parameters and FLOPs. Finally, DECDH preserves the achieved accuracy while providing the largest efficiency gain in the detection head, reducing 1.4 M parameters and 3.0G FLOPs relative to the GEIT + DIM configuration. These results indicate that the three modules are complementary: GEIT strengthens structural cues, DIM improves adaptive multi-scale representation, and DECDH ensures efficient and detail-preserving prediction.

## Conclusion

In this work, we proposed GDD-YOLO to address three coupled bottlenecks in high-resolution small object detection: loss of fine boundary cues after repeated downsampling, limited scale adaptability of fixed convolution kernels, and the computational redundancy of conventional detection heads. GEIT mitigates the first issue by extracting Sobel-based edge responses from high-resolution shallow features, constructing aligned multi-scale edge maps via progressive pooling, and injecting them into deeper backbone stages through ConvEdgeFusion, thereby providing explicit boundary priors for small-object localization. DIM addresses the second issue by dynamically aggregating lightweight convolution branches with different receptive fields according to the input response, which improves multi-scale representation without introducing heavy feature-fusion overhead. DECDH addresses the third issue by sharing convolutional parameters across pyramid levels and combining local-detail extraction with broader contextual modeling through DEConv, thus reducing redundancy while maintaining discriminative prediction features for small objects.

On the VisDrone dataset, GDD-YOLO-S achieves 26.2 AP and 42.1 AP50. Compared with YOLOv11-S, it improves AP by 2.4 points and AP50 by 2.9 points, while reducing parameters from 9.5 M to 7.9 M and FLOPs from 22.6G to 19.3G, with FPS increasing from 135 to 139. The ablation study further verifies the complementarity of the three components: GEIT alone improves AP/AP50 from 23.8/39.2 to 24.7/40.7; adding DIM raises performance to 26.2/42.0 while reducing model complexity from 11.2 M parameters and 23.4G FLOPs to 9.3 M and 22.3G; replacing the baseline head with DECDH preserves AP at 26.2, slightly improves AP50 to 42.1, and further reduces complexity to 7.9 M parameters and 19.3G FLOPs. Besides the accuracy improvement, the reduced model size, lower runtime memory footprint, and faster inference further support the practical deployment potential of GDD-YOLO in resource-constrained scenarios. These results demonstrate that GDD-YOLO achieves an effective balance between localization accuracy, scale robustness, and real-time efficiency for deployment in resource-constrained small-object detection scenarios.

Future work will focus on more general small-object representation learning under extreme scale variation and heavy background clutter, extending dynamic computation to additional stages of the detector, and evaluating cross-domain transfer to scenarios such as remote sensing and medical imaging.

## Data Availability

The data that support the findings of this study are openly available in GDD-YOLO at https://github.com/xishaoyuan/GDD-YOLO.
